# Epidermis as a Platform for Bacterial Transmission

**DOI:** 10.3389/fimmu.2021.774018

**Published:** 2021-12-01

**Authors:** Fernando Baquero, Claudia Saralegui, Daniel Marcos-Mencía, Luna Ballestero, Sergio Vañó-Galván, Óscar M. Moreno-Arrones, Rosa del Campo

**Affiliations:** ^1^ Servicio de Microbiología, Hospital Universitario Ramón y Cajal and Instituto Ramón y Cajal de Investigación Sanitaria (IRYCIS), Madrid, Spain; ^2^ Network Center for Research in Epidemiology and Public Health (CIBERESP), Madrid, Spain; ^3^ Servicio de Dermatología, Hospital Universitario Ramón y Cajal, and Instituto Ramón y Cajal de Investigación Sanitaria (IRYCIS), Universidad de Alcalá, Madrid, Spain; ^4^ Department of Health Sciences, Universidad Alfonso X El Sabio, Madrid, Spain; ^5^ Centro de Investigación en Red en Enfermedades Infecciosas (CIBER-EEII), Madrid, Spain

**Keywords:** epidermis microbiota, bacterial transmission, protection pathogens, heterogeneity transmitters, skin tribology

## Abstract

The epidermis constitutes a continuous external layer covering the body, offering protection against bacteria, the most abundant living organisms that come into contact with this barrier. The epidermis is heavily colonized by commensal bacterial organisms that help protect against pathogenic bacteria. The highly regulated and dynamic interaction between the epidermis and commensals involves the host’s production of nutritional factors promoting bacterial growth together to chemical and immunological bacterial inhibitors. Signal trafficking ensures the system’s homeostasis; conditions that favor colonization by pathogens frequently foster commensal growth, thereby increasing the bacterial population size and inducing the skin’s antibacterial response, eliminating the pathogens and re-establishing the normal density of commensals. The microecological conditions of the epidermis favors Gram-positive organisms and are unsuitable for long-term Gram-negative colonization. However, the epidermis acts as the most important host-to-host transmission platform for bacteria, including those that colonize human mucous membranes. Bacteria are frequently shared by relatives, partners, and coworkers. The epidermal bacterial transmission platform of healthcare workers and visitors can contaminate hospitalized patients, eventually contributing to cross-infections. Epidermal transmission occurs mostly *via* the hands and particularly through fingers. The three-dimensional physical structure of the epidermis, particularly the fingertips, which have frictional ridges, multiplies the possibilities for bacterial adhesion and release. Research into the biology of bacterial transmission *via* the hands is still in its infancy; however, tribology, the science of interacting surfaces in relative motion, including friction, wear and lubrication, will certainly be an important part of it. Experiments on finger-to-finger transmission of microorganisms have shown significant interindividual differences in the ability to transmit microorganisms, presumably due to genetics, age, sex, and the gland density, which determines the physical, chemical, adhesive, nutritional, and immunological status of the epidermal surface. These studies are needed to optimize interventions and strategies for preventing the hand transmission of microorganisms.

## Introduction

The transmission of infectious microorganisms between individuals through skin contact has long been known, driving the development of hygiene because of the impracticality of perpetual skin sterilization. Our knowledge of the skin microbiota has increased considerably since the introduction of massive sequencing techniques, particularly for bacteria and fungi, demonstrating the existence of specific ecosystems in differentiated areas. As with other colonized body surfaces, the detailed composition of a healthy microbiota has not yet been fully defined. Interest in bacterial transmission *via* the skin has been limited to the spread of pathogens; however, most skin microorganisms can be classified as both commensal and pathogenic, as is the case for *Cutibacterium acnes*, which is commensal in almost all patients but also causes acne (1).

Skin microbiota can be transmitted by direct contact but is also released into the atmosphere through the desquamation process. The environment is an almost infinite source of microorganisms suspended in the air and water systems or deposited on surfaces (2), where individuals exchange microorganisms as donors and recipients. The current SARS-CoV-2 pandemic has highlighted the importance of determining certain microorganisms’ skin transmission ability (3), particularly the high transmission between individuals in enclosed and crowded spaces such as public transport and swimming pools (2, 4, 5). After being transmitted, the foreign microorganism can colonize the skin, sometimes causing an infection. The epidermis also acts as a platform for transmission to other individuals/environments. While differences in bacterial transmission capacity have been extensively studied, there are still unknown human factors favoring or limiting the transmission of exogenous bacteria by the skin.

Continuous external microbial exposure ensures the frequently transient diversity of the skin microbiome. In the hospital setting, the skin microbiome’s (both for patients and healthcare workers) should be carefully controlled, as it is a key source for transmission events involving pathogenic bacteria. In fact, nosocomial infection often follows skin colonization by antibiotic multiresistant pathogenic bacteria. Hand washing by healthcare workers is still the best strategy for preventing the transmission of nosocomial pathogens, as has been demonstrated for *Clostridioides difficile* whose spores are resistant to the action of alcohol gels and standard disinfectants (6) and should be eliminated by shedding through standardized structured washing techniques (7).

## Epidermis, the Border With the Microbial Environment

Our epidermis, the outermost side of the skin that covers 2 m^2^ on average, is not only exposed to a multiplicity of environmental organisms but also repeatedly makes contact and rubs against contaminated natural and artificial surfaces. The skin is a compartmentalized habitat, with specific microecological spaces such as keratinized space, sebaceous glands, and apocrine and eccrine glands where the bacterial density can differ considerably. There is also a critical epidermis-mucosal border, where mucosal microbial populations can coalesce with those established on the epidermal surface.

Despite the considerable increase in recent years in our knowledge of human microbiota, more investigation is needed into the interactions between the two microbial worlds of muco-cutaneous junctions, from the point of view of biochemistry, microecology, and immunology (8). It seems clear that there is no sharp border but rather a gradient of conditions, likely dependent on physical variables such as humidity and temperature. These transitional areas are obvious in the lips, where we observe the non-keratinized epithelium of the labial mucosa transitioning to the buccal mucosa, with surfaces changing with age (9–11). Similar “borders” occur at the eyes, rectum, and vaginal mucosa and the neighboring keratinized epidermis. The microbiota at these borders has been poorly characterized.

The common use of fingers for exploring and washing mucosal orifices and during sexual activities is another important source of interactions between epidermal and mucosal microbes. Artificial mucosal-epidermis interfaces are frequently created in surgery (e.g., in ileostomies), sometimes with pathogenic consequences (12). In contrast, certain less exposed skin regions, such as interdigital and other skin folds, could constitute a potential healthy microbiome reservoir for re-colonization of altered epidermal communities.

The microbial dialogue with the skin’s immune system, both with the innate and adaptive cells, determines the tolerance or inflammation response. Although the response to microbial infections is well known, the determinants of a tolerance status are not. Dermatological research is currently focused on the interplay between the immune system and skin microbiota for diseases such as acne and seborrheic dermatitis (13). In these skin conditions, the pathogenic process is probably based on an excessive response of the local innate immunity against members of commensal microbiota, including bacteria such as *Cutibacterium acnes*, fungi, as *Malassezia furfur*, and viruses, as Merkel cell polyomavirus or herpesvirus.

A proinflammatory gut microbiota has been reported in patients with alopecia areata universalis (14), and there have been supporting epidemiological hypotheses such as the relationship between rosacea and Parkinson’s disease on one hand and the skin-gut-brain axis on the other (15). Detailed studies of the local microbiome, such as the follicular microbiome, provide valuable information on the etiopathogenesis of chronic inflammatory diseases such as primary cicatricial alopecia and facilitates their understanding and classification (16). There has recently been major interest in microbiota composition as a predictor of drug response, particularly for the gut ecosystem and immunotherapy in melanoma, and it has been proposed the transference of fecal microbiota from immunotherapy responders to non-responders (17, 18).

## The Epidermal Microbiome and Modulation Strategies

Is the epidermal microbiome representative of the dermal microbiome? **Table 1** shows the bacterial genera which are shared in the dermal and epidermal compartments. In general, bacterial composition of the stratum corneum does not significantly differ from that of the full skin (20, 21). Sampling by swabbing therefore yields analogous results to sampling by tapping (22). The “constant” epidermal microbiome is a minority subset of the dermal microbiome, which constitutes a “real microbiota”, extremely stable and universal in human hosts (19), where bacteria are adapted to the nutritional conditions of the dermal compartment. However, the shared bacteria are much more abundant in number in the epidermis, which also contains a high density of diverse and transient bacterial organisms, as expected by the frequency of environmental contacts, and consequently variable among individuals. Given that our focus is on transmission, we will not go into detail on the detailed skin microbiome composition, a topic that has been covered by other authors (21, 23, 24).

**Table 1:** Bacterial microbiota in the epidermal and dermal compartments.

Epidermal-Dermal Genera Not Phylum Proteobacteria	Epidermal-Dermal Genera Phylum Proteobacteria	Epidermal-Dermal Genera Anaerobes
***Corynebacterium***	***Pelomonas***	*Finegoldia*
***Staphylococcus***	***Acinetobacter***	*Peptoniphilus*
***Micrococcus***	***Moraxella***	***Anaerococcus***
***Streptococcus***	*Pseudomonas*	*Blautia*
***Paracoccus***		*Porphyromonas*
***Brachyobacterium***		*Fenollaria*
*Kocuria*		***Veillonella***
*Dietzia*		*Cylindrospermum*
*Actinomyces*		*Prevotella*
*Brevibacterium*		*Dialister*
*Tepidimonas*		*Bifidobacterium*

This Table was inspired by the publication referenced as Bay et al. (19).

Epidermal microbiota is much more abundant and variable (strongly affected by environmental contacts) than dermal microbiota. A subset of most frequent members of the epidermic microbiota constitute a very stable and universal (preserved in different individuals) dermal bacterial community, adapted to the nutrients of the dermal compartment. In the boxes below, listed by frequency, the epidermal genera with high representation in the dermal compartment are highlighted in bold characters.

At birth, single clones of a variety of microorganism colonize human skin. The environmental pressures and particular conditions experienced by the individual lead to microevolutions that result in lineage diversification, even in commensal *S. epidermidis* (25). During diversification, commensal microbiota can experience genetic acquisition or loss after punctual interactions with the transient transmitted bacteria. In addition, virulent and antibiotic-resistant clones, such as the Panton-Valentine leukocidin-producing methicillin-resistant *S. aureus* clone USA300, are habitually transmitted by skin contact and cause major outbreaks (26, 27).

The pathogenicity of particular *C. acnes* lineages has been reported not only in acne but also in systemic diseases such as prostatitis/prostate cancer, synovitis-acne-pustulosis-hyperostosis-osteitis syndrome, sarcoidosis, sciatica, and implant-associated infections. Skin microbiota transplantation from a healthy donor has been proposed to treat dermatologic conditions (28, 29), mainly to eradicate virulent *C. acnes* and *S. aureus* clones, or at least to replace the bacteria with non-virulent ones. The utility of bacteriophages for skin infections, particularly those caused by *Pseudomonas* in extensive burns, has recently been recently reviewed (30).

## Bacterial Nutrition and Growth on the Epidermis

Lipids excreted by sebaceous glands (frequently in the facial epidermis) contain anti-bacterial substances and protective compounds and are a nutrition source (31). Gram-positive bacteria in the skin, mainly *Staphylococcus* and *Cutibacterium*, release exoenzymes to enhance the recovery of nutrients from the environment, particularly proteases for amino acid liberation from skin proteins such as keratins, collagen and elastin (32). For instance, lipase production for triglyceride lipid degradations is significantly higher around comedones, causing inflammation in acne (21, 33). Other exoenzymes include bacterial hyaluronidase, which enable the obtention of glucuronic acid and N-acetyl-D-glucosamine from long-chained hyaluronic acid, and DNase, which likely degrades the extracellular DNA from apoptotic keratinocytes or corneocytes. *C. acnes* can induce keratinocyte autophagy by stimulating the CD36-CD14-TLR2/4-TLR6 signaling module, triggering ROS generation through nicotinamide adenine dinucleotide phosphate oxidase and the TRAF6-ECSIT-NLRX1 pathway and evoking mitochondrial dysfunction (34, 35). However, we still lack the full picture of microbial nutrition in the skin, particularly regarding the role of protocooperative actions among bacterial species in nutrient exploitation.

The critical factors of bacterial nutrition and growth in the epidermis are pH and water availability, which also determine the concentration of free amino acids and lactate. For example, the water content of the stratum corneum of Japanese individuals (as measured by Raman spectroscopy) ranges from 30% at the surface to 70% in the deeper layers (36). Prolonged water exposure significantly increases the epidermal water content; however, the external part of the stratum corneum gradually dries out after the forced hydration is discontinued (36). The water content of the skin surface is then mainly based on excretion by eccrine sweat glands. However, free amino acids, glycerol, sphingolipids and particularly metabolites of filaggrin, a large protein bound to lipids and keratin, such as urocanic acid and pyrrolidone carboxylic acid, also function in the outer skin as natural moisturizing factors, able to absorb large amounts of water and maintain a low pH (37). The epidermal water content depends on age, anatomical site and season. Certain molecules (such as teichoic acids) on the external surface of Gram-positive bacteria can produce local proinflammatory effects (38), which results in greater water availability resulting from increased vascularity. Osmolarity increases with water paucity. Water tends to be retained in the epidermal keratinocytes outer layer, particularly in the valleys where it is protected from evaporation; in addition, under osmotic stress more water-channels are produced, as aquaporin-3 (39). The skin’s retention of transmitted bacteria depends strongly on water (40). Sweat increases water availability, transports molecules with bacterial impact, and facilitates bacterial transmission through signals contained in their extracellular vesicles.

## Bacterial Death on the Epidermis

Keratinocytes contribute to the innate immune response system by sensing microbial cell density by detecting pathogen-associated molecular patterns (PAMPs) through pattern recognition receptors (PRRs) (21). Such detection results in the induction of release of cytokines, chemokines, and antimicrobial peptides (AMPs) such as human beta-defensins HBD-1, HBD-2, and HBD-3, cathelicidin LL-37, and the antimicrobial proteins RNase 7 (from the RNase A superfamily) and Perforin-2, the last being able to kill intracellular organisms (41–43).

**Figure 1:**
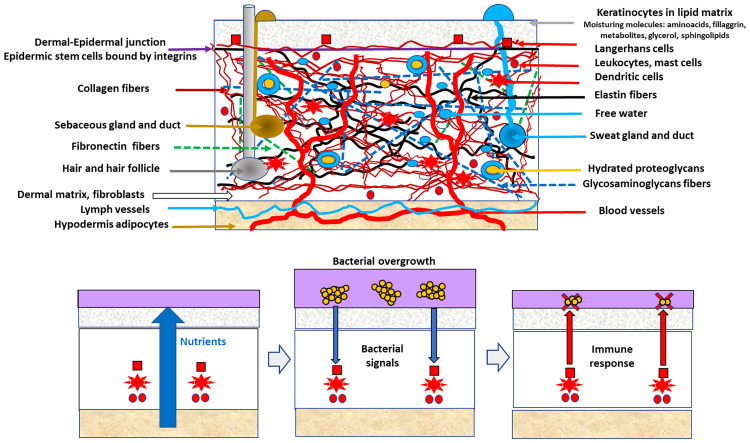
The hypothesis of indirect epidermal bacterial clearance of a pathogen. Up in the figure, a schematic view of the skin is presented, particularly of the components of the dermal compartment, determining the commensal microbiota of the epidermis. Below, the indirect epidermal bacterial clearance of a pathogen (clusters of yellow circles) in the violet layer (surface commensal microbiota). Conditions facilitating the growth of commensals (vertical blue thick arrow) also favors pathogens, but the commensals overgrowth stimulate (narrow blue arrows) the defense system based on cellular local immune innate response involving cellular (red squares, circles, stars) and glands secretion. Antimicrobial defense (red arrows) reduces the overall bacterial density, but the lower-numbered pathogens are eliminated (red X symbol), whereas the dominant commensal population is reduced to its normal density (sequence from the second to the third box at the bottom).

The response is modulated by molecules on the surface or those released by commensal organisms (44). The conditions favoring the overgrowth and immunological tolerance of skin commensals are frequently those that are also beneficial for the growth of certain skin pathogens (**Figure 1**). The “bacterial overgrowth” signal is therefore likely triggered by commensals, resulting in decreased commensal density; however, this decrease is deleterious for less numerous pathogens. The overgrowth of skin commensals likely releases immunity-stimulating signals, including structural bacterial molecules such as teichoic acids or proteins released from the bacterial cell wall, microbial-secreted substances such as porphyrins, and molecules, as oleic acid, resulting from the bacterial metabolism of local lipid substrates (45). Overgrowth is eventually followed by intracellular engulfment and the release of more signaling molecules such as complement and interleukin-1. Perforin-2 upregulation following *S. epidermidis* overgrowth increases the intracellular killing of *S. aureus* (41). The innate lymphoid cells regulate the production of antimicrobial lipids (such as palmitoleic fatty acids), reducing the population density of *Staphylococcus* and, in general, all Gram-positive bacteria (46).

In normal conditions, a “normal”-sized population of commensals (such as *S. epidermidis*) stimulates only the innate defense system (47). Pathogens such as *S. aureus* have evolved mechanisms for subverting immune stimulation, such as modifying their PAMPS (48); however, these bacterial protection mechanisms are expected to be effective only after reaching a cell-density threshold.

The strategy of eliminating pathogens by regulating commensal overgrowth (**Figure 1**) is based on the differences between highly adapted organisms (commensals, those with large populations) and less adapted organisms (occasional pathogens, those with small populations) that compete for the same resources (47). Commensals are better endowed to reconstruct the original population density after a challenge. In fact, the population density of commensals is by itself a limiting factor for pathogenic colonization, either indirectly (e.g., by nutritional competition) or directly (e.g., antagonistic substances). Direct antagonism between commensal and pathogenic staphylococci is frequently mediated by secondary metabolites such as low molecular weight bacteriocins, frequently of the lantibiotics type, the equivalent to microcins in intestinal Enterobacterales (49, 50). Lantibiotics released from commensal *Staphylococcus* are synergistic with the human cathelicidin antimicrobial peptide LL-37 in reducing *S. aureus* populations (51). *S. epidermidis* might also interfere with *S. aureus* biofilm-type colonization by expressing the serine-type protease Esp. In fact, protection against *S. aureus* involvement in atopic dermatitis by increasing the density of commensal microbes is being explored. The immunological response against pathogens might in fact depends on resident commensals. The shedding of the stratum corneum acts as a sink for microorganisms adhered to the skin surface; however, these organisms persist by colonizing the deeper layers of the epidermis thereby keeping the population density stable.

## Gram-Positive *Versus* Gram-Negative

The conditions of low moisture, high osmolarity, low pH, and AMPs presence clearly favors organisms with a thick peptidoglycan envelope and lacking an outer membrane, as well as Gram-positive organisms, with the interesting exception of *Enterococcus* species, which are not usually found in the epidermis. Gram-negative organisms are not permanent colonizers of the outer skin, with the exception of *Acinetobacter* in moist intertriginous areas. In experimental transmission studies, Gram-positive organisms, such as *E. faecium* and *S. aureus*, exhibited the highest transmission efficiency, whereas Gram-negative organisms were less efficient, particularly *E. coli*, which had the lowest transmission rate (52). The recovery of phages from Gram-negative *Proteobacteria* might simply reflect the occasional, transient colonization of these microorganisms, with the possible exception of individuals with primary immunodeficiencies (53, 54). However, some commensal species from the Phylum Proteobacteria, as *Pelomonas*, are consistently present in the dermal compartment, and might migrate to the epidermis (**Table 1**). Important potential human pathogens such as *Enterobacterales* and *Enterococcus* are rare in normal epidermis, despite expected frequent external contamination from the intestinal microbiota. It is certainly plausible that Gram-positive commensals might elicit the production of human antimicrobial peptides able to eradicate small populations of Gram-negative bacteria, using the strategy illustrated in **Figure 1**.

Skin osmolarity also favors Gram-positive bacteria. Certain *Proteobacteria* can be detected by 16S rDNA sequencing from the skin surface, including *Acinetobacter, Enterobacter, Klebsiella, Pseudomonas, Serratia* and *Sphingomonas*, all of them environmental organisms and likely transient “skin-landing” bacteria. In short, the epidermis should not be considered a niche for *Enterobacterales* or *Enterococcus*, given that it does not support the significant growth of these critical human pathogens (55). If these organisms are just transiently bound to the epidermis by weak unspecific adhesion, the possibility of being transmitted between hosts could be enhanced.

A rarely considered aspect of the epidermal bacterial microecology (and transmission) is the spatial structure of bacterial cells, ranging from homophilic clumps to larger biofilms. In particular, *Staphylococcus* tends to clump into multicellular aggregates (staphyle is the Greek name for a bunch of grapes), facilitating adhesion to the skin and likely colonization. The intercellular homophilic bonds of *Staphylococcus* depend on a polycationic polysaccharide intercellular adhesin; however, the role of cell wall-anchored proteins (self-aggregating molecules) appears to be critical in the process. These proteins (e.g., the bacterial surface serine-aspartate repeat protein SdrC) might also be considered a multifunctional adhesin involved in hydrophobic interactions with surfaces. In fact, clumping-negative *S. aureus* mutants are less adhesive to surfaces, including keratinocytes (56).

The interaction between bacteria and the colonizable surfaces is mediated by cell appendages and specific molecules located at the cell surface. In particular, a large variety of adhesins assures the close contact required to exploit structured environments. Many of these adhesins are involved in the attachment to the host tissues including skin, and also contribute to in the formation of interbacterial interactions resulting in the formation of local biofilms (bacterial multicellular aggregates), increasing the resilience (permanence) of adhered bacterial populations. The molecular determinants mediating specific bacterial adhesion to the outer layers of the epidermis remain scarcely investigated for most bacterial taxa. On the contrary, excellent information is accessible for some pathogenic Gram-positive bacteria, including a thematic Research Topic in Frontiers in Microbiology (57). Seminal reviews are available on *Staphylococcus* adhesion mediated by surface proteins, mostly comprising the hydrophobic LPXTG transmembrane motif assuring cell-wall anchoring. Many of these surface components recognize adhesive matrix molecules as fibronectin, vitronectin, laminin, fibrinogen or elastin, the MSCRAMM family. Fibronectin-binding adhesins (FnBPA and FnBPB in *S. aureus*) regulated by *agr* and *sar* operons, are critical in the adhesion to the squamous epithelium (58–61).

Interestingly, *Staphylococcus* LPXTG proteins and some extracellular adherence proteins also promote bacterial internalization by keratinocytes, assuring the permanence of the bacterial population in the epidermal compartment (62). Populational resilience is enhanced by biofilm formation, involving, among others, Aap and SdrF proteins (63). Interbacterial adhesion on the epidermis is also favored by the small basic protein (Sbp). This protein favors the formation of amyloid fibrils structuring the biofilm matrix derived from the effect of adhesins, comprehending microbial polysaccharide (mostly β-1-6-linked N-acetylglucosamine), intercellular adhesin surface proteins, DNA, and proteins from dead cells (60). Other surface components of Gram positives, as teichoic acids, also contribute to skin adhesion (64).

Consider that in *S. aureus* there are 24 different LPXTG adhesins with different specificities and roles. *S. epidermidis* expresses more than 10 adhesins (60, 61). *Staphylococcus lugdunensis* is also a frequent commensal in the outer layers of the skin and contains the LPXTG protein SrtA (65, 66).

In the case of *Cutibacterium* bacteria, mostly probably originated in the sebaceous glands-ducts where they have their niche (67, 68), they migrate to the epidermis and remain attached by their fibronectin-binding surface proteins, also inducing keratinocytes internalization, which assures the local permanence (35, 62).

The skin adhesion of bacterial spores, including those of pathogenic organisms such as *C. difficile*, is poorly known, but approximately 5% of hospital health workers are carriers of these organisms (69, 70). Future advances in the “ecology of adhesion” might result from applying confocal laser scanning microscopy, given that it has been used for human mucosal surfaces (71).

## The Role of the Epidermis in Transmitting Human Pathogens

Although certain key human pathogens are not permanently established on the surface of the normal epidermis, they use the surface as a platform for host-to-host transmission. In the case of *Enterobacterales*, the hands of intensive care unit (ICU) staff are frequently (nearly 20%) contaminated by the same *Klebsiella* clones that infect patients; however, the absolute count by culture does not exceed 10^3^ per hand, and the survival time was estimated at approximately 2–3 h (72). In fact, in abiotic dry surfaces, such as computer keyboards in the ICU used by nurses and doctors, only Gram-positive skin bacteria are recovered, mostly *S. epidermidis* (73). As in the case of ICU health workers, environmentally linked individuals tend to share bacterial organisms. In fact, easy transmission of antibiotic-resistant *Enterobacterales*, presumptively by hand contamination, occurs among household members and individuals who visit friends and relatives (74–76). In closed communities, a common community epidermal microbiota is expected.

Most of the published works on the epidermal transmission of human pathogens are derived from hand-washing studies, one of the key “rituals” in preventive medicine. Although the efficacy of hand washing is extremely difficult to estimate, hand washing is undeniably highly effective in decreasing the risk of contaminating sterile tissues, mechanical devices and food by low-numbered pathogenic bacteria, as shown in the seminal work by Semmelweis (77). The effectiveness of hand washing in eliminating microbial organisms is inversely proportional to the skin’s bacterial load and frequently has only a marginal, transient effect on heavily inoculated fingertips (78). It is noteworthy that Semmelweis discarded as irrelevant the use of microscopes to explain his results (77). That resulted in an epistemologically utilitarian “Semmelweis Complex” recommending that the focus be on efficacy and not the scientific reasons for explaining the effect. Such scientifically deleterious Complex remains very much alive in public health. To quote a modern reference, “studies on practical and efficient means to increase compliance with hand hygiene guidelines and to influence behavior surely are needed more than are elaborate and sophisticated studies on the effects of hand washing” (79). Such generalized “practical” view has delayed experimental studies, and consequently there are numerous issues in hand-washing biology that are unknown or poorly understood. For instance, if the hand’s commensal bacteria can prevent the growth of potential pathogens, the controlled reduction of these commensals might be more advisable than full eradication (80). Increasing our knowledge of the biology of epidermal bacterial transmission appears to be the only option for improving our interventions.

## Experimental Bacterial Transmission

The biology of hand-to-hand transmission is a recent field of research. In a seminal study published in 2014 (81), broth suspensions of potentially pathogenic well-defined clones of *E. faecium*, a non-skin commensal Gram-positive organism, were gently deposited and spread on both thumb tip surfaces (approx. 10^7^ cells over 1.32 cm^2^) of 30 healthy volunteers (4 experiments per individual, spread over 6 months). After complete drying, the organisms from a sample surface of 0.78 cm^2^ on one of the thumbs were suspended by shaking them in a saline solution. The second contaminated thumb was put in close static contact (with minimal pressure and preventing twists or wipes) with the index fingertip of the other hand of the same individual for 10 s, and the transmitted organisms were also suspended. Over several steps involving the fingers of both hands, a quantitation curve of the proportion of finger-to-finger transmission was obtained. The bacterial count showed an exponential decay in sequential finger-to-finger transfers in most of the volunteers, typically a decay of 1.5 log in successive counts. This result is consistent with that obtained in similar experiments on skin bacterial survival and hand transmission (82–84). Interestingly, in the *E. faecium* experiment, the frequency distribution of the exponential decay parameter estimated for all individuals clearly showed an asymmetrical right tail containing an overrepresentation of high transmitter individuals (13% of the volunteers) with their epidermis exponential decay parameters close to zero. A variable transmission rate of the various *Enterococcus* clones was also observed, supporting the suggestion that a number of bacterial variants (including Gram-negative bacteria, such as *Proteobacteria*) could be better adapted than others to the environmental skin circulation (81, 85). A further analysis of the data proposed 3 transmission efficiency categories: poor, medium, and high finger-to-finger bacterial transmitters. All 10 male volunteers were classified as poor or high intrahost transmitters, whereas almost all 20 of the female participants were grouped in the medium category (53).

Experimental fingertip transmission studies extended to interhost transmission have shown that the success of the transmission chain depends on the position of a poor transmitter in the series. Poor-transmitter volunteers had the ability to cut off the transmission chain independently of their position (53). Certainly, these preliminary observations might foster further research to determine the individual risks of foodborne transmission and healthcare workers.

## The Basis for Individual Variability in Bacterial Transmission

The density and diversity of skin commensal bacteria might affect the fate of pathogenic bacteria contacting the surface. In contrast to the low variability of dermal microbiota across humans, the epidermis shows a higher polymorphic set of bacterial species, probably because they reflect the host’s lifestyle and changing environment (e.g., altitude or temperature) (19, 86). Interindividual genetic differences in epidermal microbes cannot be totally ruled out, given that variations occur with organisms in fish skin and that of sea mammals (87, 88). In humans, these genetic differences might occur (45, 89, 90), but the effects of race have not been sufficiently evaluated, although peculiarities of the skin microbiota of Chinese populations have been suggested (90). Genetics and sex can affect the density of eccrine, apocrine, and sebaceous glands determining the physical, chemical, adhesive, nutritional, and immunological status of the epidermal surface and thereby the density and type of organisms. The influence of age, stress, and hormonal status also cannot be overlooked. In the above-mentioned experiments regarding *E. faecium* finger-to-finger transmission, no significant association for transmission decay was found between fingertip temperature measurements and finger pressure (53, 81). There is a need for studying the intraindividual and interindividual transmission of organisms from various epidermic sites, such as sebaceous areas (e.g., face and back), moist areas (e.g., the armpits and the webbed parts of the fingers/toes), dry areas (e.g., forearms and buttocks), and sites containing varying densities of hair follicles, skin folds, and skin thicknesses. The role of epidermal friction ridges is a possible line for further research into the biology of the epidermal transmission of bacteria.

## Tribology of Skin: Friction Ridges on the Fingertips

**Figure 2:**
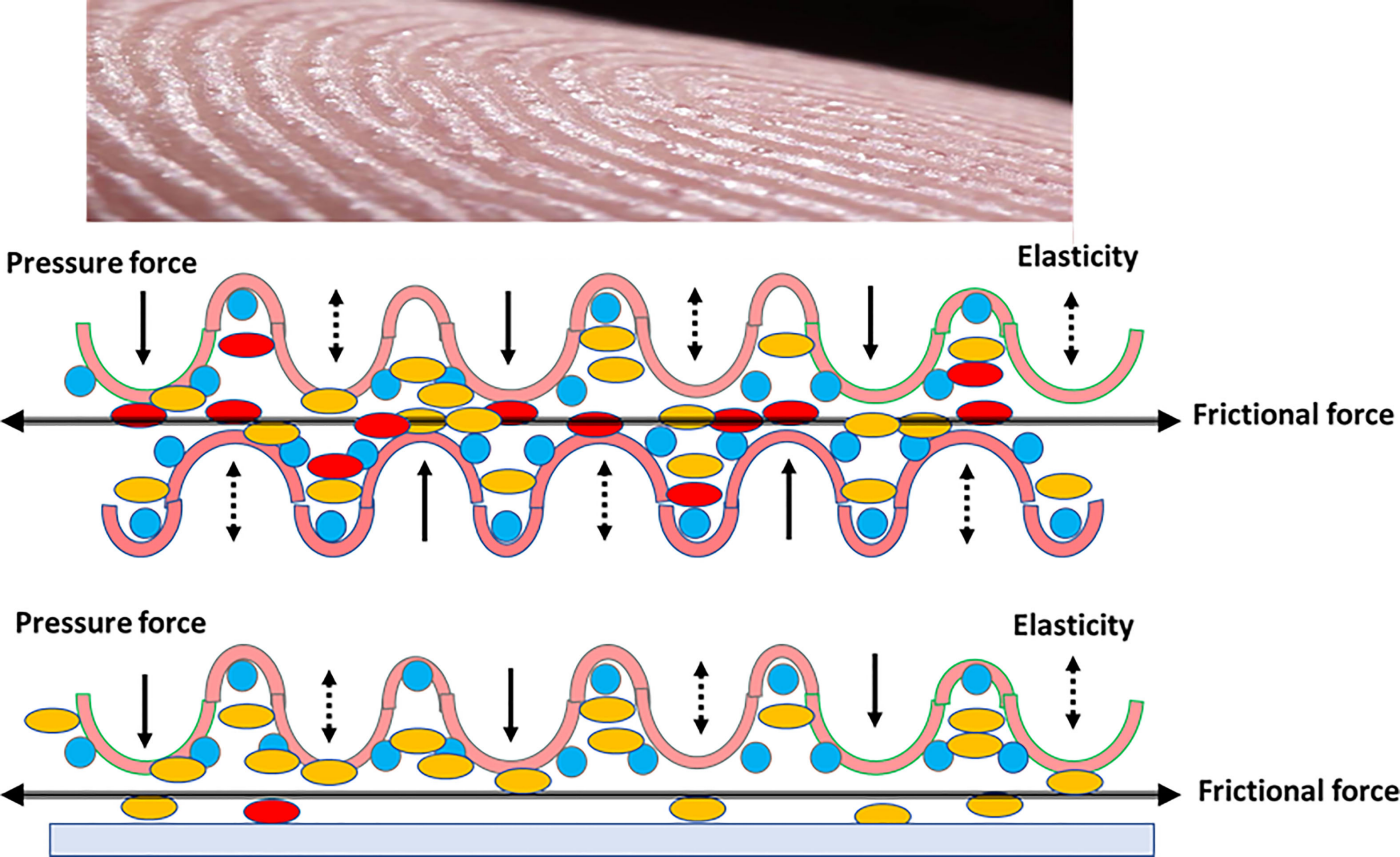
Epidermis tribology. Up in the figure, the surface of a finger pad, showing the epithelial pad crests. Below, two rubbing series of friction (beheaded arrow) finger pad crests. Because of the pressure and frictional forces (black arrows), and elasticity of the dermal compartment, the (yellow) bacteria of the upper epithelium frequently migrate to the lower epithelium, and only some of the (red) bacteria are transmitted to the green epithelium. The asymmetry of transmission might be due to microecological differences, for instance water content (blue circles). Down in the slide, if the epidermis is rubbed on a smooth surface, the transmission is much less effective.

Tribology is the science of interacting surfaces in relative motion, including, as said before, friction, wear and lubrication. Tribological phenomena occur on a large scale, and include microbiotribology, as in the case of homophilic bacterial cell interactions, giving rise to frictional phenomena, possibly creating electromagnetic fields that influence collective and individual cell behavior (91, 92). For human surfaces, a greater understanding is needed of the nonlinear effects of plasticity, adhesion, friction, wear, lubrication and surface chemistry, all of which play a part in skin tribology (**Figure 2**) (93).

Fingertips, which are critical surfaces in human-to-human bacterial transmission, are covered with friction skin (94, 95). Papillary ridges in friction skin likely evolved to assist in grasping and holding onto objects and are important in the biology of primates and koalas (96). The equilibrium between friction ridges and optimal hydration (eccrine glands) of the keratin layer maximizes friction (97). In humans, skin friction and microbial transmission depend on the tribological configuration; the surface roughness and the finger pad sweat rates. Age modifies friction forces by reducing skin thickness, dermal elasticity, and by ridge flattering, possibly in relation with collagen reduction (98). Dynamic optical coherence measurements by tomography will likely be essential for determining the frictional behavior of human finger pads (99, 100). We expect these studies to be applied soon to the human-to-human transmission of microorganisms (101, 102). Nothing is known about the triboelectric effects of ridge friction (which creates magnetic fields) on bacterial adhesion and repulsion. Multivariate models for predicting the frictional behavior of human skin, thereby identifying “high-transmitter” populations (81), could have obvious applications in public health, from food microbiology to hospital-based cross-infections.

## Author Contributions

FB and RdC wrote the manuscript; CS, DMM and LB provided information on microbiology, SGV and OMMA supervised and provided dermatological information. The final manuscript is approved by all authors. All authors contributed to the article and approved the submitted version.

## Funding

This work was supported by the Instituto de Salud Carlos III PI20/00164 to RdC, and REIPI (RD16/0016/0011) actions, cofinanced by the European Development Regional Fund “A way to achieve Europe” (ERDF). FB was supported by InGEMICS-CM (B2017/BMD-3691), funded by Comunidad de Madrid (Spain) and European Structural and Investment Funds; and by the CIBER in Epidemiology and Public Health, CIBER-ESP (CB06/02/0053), integrated in the Spanish 2013-2016 I+D+i State Plan and funded by Instituto de Salud Carlos III.

## Conflict of Interest

The authors declare that the research was conducted in the absence of any commercial or financial relationships that could be construed as a potential conflict of interest.

## Publisher’s Note

All claims expressed in this article are solely those of the authors and do not necessarily represent those of their affiliated organizations, or those of the publisher, the editors and the reviewers. Any product that may be evaluated in this article, or claim that may be made by its manufacturer, is not guaranteed or endorsed by the publisher.
